# Supplementary material for: Nutrition and Physical Activity as Modulators of Osteosarcopenic Adiposity: A Scoping Review and Recommendations for Future Research
Source: Nutrients. 2023 Mar 27;15(7):1619. doi: 10.3390/nu15071619 (PMC10096523; doi:10.3390/nu15071619)
Supplement: Supplementary file 1 [file nutrients-15-01619-s001.zip › nutrients-2289667-SI.pdf]

Table S1. Characteristics and results of studies with dietary intake and/or nutritional status as independent variables related to osteosarcopenic adiposity/obesity (OSA/OSO)[1]

Reference, Studied topic	Country, Setting	Study Design	Diagnostic criteria & Instruments			Sample size, *n* (%) Intervention	Age (years)	OSA/OSO Prevalence[2] *n* (%)	Assessment Tools	Compared to[3]	Outcomes in OSA/OSO group (or others if indicated)
			Bone	Lean/Muscle	Adipose						
Cvijetic, S, 2023 [16], Nutritional status in nursing homes residents during COVID	Croatia, Six Nursing Homes	C-S Inclusion/ Exclusion criteria applied	T-score ≤-1 for total bone mass With BIA-ACC	S-score ≤-1 With BIA-ACC	BF%: F ≥32; M ≥25 With BIA-ACC	Total, n=365; F, n=296 (81); M, n=69 (18.9)	Mean, 83.7 F, 84.3 M, 83.1	Total, n=242 (66.3); F, n=209 (70.8); M, n=33 (47.8)	BIA-ACC BioTekna® Mini Nutritional Assessment (MNA); Other questionnaires	Normal; Others, combination of: osteoporosis and/or sarcopenia and/or obesity alone	-32.4% and 31.3% of F and M were at risk for malnutrition and 5.8% and 6.2% of F and M, respectively were malnourished;<br>-No difference in malnourishment or risk of it in those with or without OSA;<br>-No difference in OSA prevalence or nutritional status in those with or without COVID;<br>-Lower phase angle (indicating lower cell integrity and muscle quality);<br>-Lower total bone mass;<br>-Higher intramuscular adipose tissue
Keser, I, 2021 [17] Several nutrients; Body water distribution in nursing home residents	Croatia, Nursing Home	C-S Inclusion/ Exclusion criteria applied	T-score ≤-1 for total bone mass With BIA-ACC	S-score ≤-1 With BIA-ACC	BF%: F ≥32; M ≥25 With BIA-ACC	Total, n=84; F, n=69 (82); M, n=15 (18)	Mean 83.5 Range 65.3-95.2	Total, n=45 (53.6); F, n=37 (53.6); M, n=8 (53.3)	BIA-ACC BioTekna® 24-h recall; Other questionnaires	Osteopenic adiposity, adiposity alone	-Lower trend for protein, omega-3, fiber, Ca, Mg, K, vitamins D and K intake;<br>-All participants consumed nutrients below recommendations;<br>-Signif. higher extracellular water, indicating higher inflammation
[NoP]Ilich, JZ,* 2019 [23] Weight loss with low fat	United States, Community dwelling	Longitudinal Inclusion/ Exclusion	T-score ≤-1 for hip and/or spine for	Total lean mass (kg); Android lean (kg)	BF%: Average at baseline 45.9	At baseline with complete data, n=135	Mean 55.8 at base-line;	Not reported; All three body	iDXA; Routine lab equipment and ELISA	Baseline values;	-All participants lost ~4%, ~3%, and ~2% body weight, fat, and lean mass, respectively;

dairy foods and calcium/ vitamin D supplements effects on bone and body composition	Caucasian, overweight/ obese postmeno-pausal women	Criteria applied; 6-month interventi on with 3 randomize d groups (dairy, suppl., placebo); All samples blinded for analysis	osteopenia (no osteoporosis) With iDXA	Gynoid lean (kg) With iDXA	With iDXA	(dairy, n=64, Ca/vit. D suppl., n=62, placebo, n=62); At 6-month, n=97 (dairy, n=32, Ca/vit. D suppl., n=37, placebo, n=30); Moderate energy restriction (85% of total energy needs) to all participants Dropout: 28.2%; Imputed analyses for missing data	6.6 years since meno-pause	composition components were measured and evaluated at baseline and after 6 months of intervention	(for blood and urine samples; 3-day dietary records; Activity records	Groups after 6 months of intervention	-<u>Dairy group</u>: signif. higher loss in waist, hip, and abdominal circumferences and body fat (total, android); signif. lower loss in lean mass (total, android);<br>-<u>Supplement group</u>: signif. lower decrease in total body, spine, radius BMD; signif. increase in femoral neck and total femur BMD
[NoP]Ilich, JZ,* 2022 [24]; Secondary analysis to Ilich, JZ 2019 [23] Weight loss with low-fat dairy foods and calcium/ vitamin D supplements effects on cardio-metabolic risk											-<u>All participants</u> improved in (due to weight loss): cardiometabolic indices (BP, TC, triglycerides, insulin, leptin, adiponectin, ApoA1, ApoB)<br>-<u>Dairy group</u>: Signif. decrease in BP, TC, LDL-C, TC/HDL-C, ApoB, leptin; signif. increase in adiponectin, ApoA1<br>-<u>Supplement group</u>: Signif. decrease in BP, triglycerides, LDL-C, ApoB, leptin; signif. increase in HDL-C, adiponectin, ApoA1
[A]Choi, M, 2021 [18] Dietary Calcium and phosphorus intake	S. Korea, KNHANES 2008-2011	C-S Retro-spective Inclusion/ Exclusion criteria applied	T-score ≤-1 for hip and/or spine to include osteopenia & osteoporosis With DXA	SMI F ≤5.4 kg/m^2; M ≤7.0 kg/m^2 With DXA	BF%: F ≥32; M ≥25 With DXA BMI: kg/m2 overweigh t ≥23<25, obese ≥25	Total, n=7007; F, n=3864 (55.1); M, n=3143 (44.9)	Mean, 62.3 OSA-65.5; More women (68.4%)	Total, n=763 (10.9) F and M combined	DXA 24-h recall	Total of 8 groups: Normal and combinations: osteoporosis, and/or sarcopenia and/or obesity alone	-Lower calcium intake signif. associated with osteosarcopenia and OSA;<br>-Lower phosphorus intake signif. associated with sarcopenic adiposity;<br>- Ca/P ratio (below median) signif.

											associated with osteopenic adiposity<br>-Signif. lower activity in OSA compared to normal group
[A]Choi, M, 2020 [19] Protein intake: total and plant-based	S. Korea, KNHANES 2008-2009	C-S Retro-spective Inclusion/ Exclusion criteria applied	T-score ≤-1 for hip and/or spine to include osteopenia & osteoporosis With DXA	ALM/Weight <1SD of Korean reference population (20-39 y old) With DXA	BF%: F ≥32; M ≥25 With DXA BMI: kg/m2 overweight ≥23<25, obese ≥25	Total, n=1351; F, n=706; M, n=645	Mean 60.5; F-OSA 65.5 M-OSA 63.8	Total, n=865 (64.0); F, n=649 (91.9); M, n=216 (33.4)	DXA 24-h recall	Normal, only; No other groups were considered	-M >65 y consuming <0.91 g/kg of protein (Korean recommend.) had 5.8 higher odds of developing OSO;<br>-Plant-based protein intake in M-OSO was higher than in M-normal.<br>-Energy consumption in M-OSA higher than in M-normal.<br>-Signif. lower intense physical activity in M-OSO
Bae, Y-J, 2020 [20] Fruit intake, vitamin C, potassium	S. Korea KNHANES 2008- 2010	C-S Retro-spective Inclusion/ Exclusion criteria applied	T-score ≤-1 for hip and/or spine to include osteopenia & osteoporosis With DXA	ALM/weight <1SD of reference population	Waist circumference ≥85 cm	Total, n=1420 F only	Range 50-64; OSO 58	n=194 (13.7)	DXA, 24-h recall	Normal; osteopenia/ osteoporosis; sarcopenia; and/or obesity	-Signif. lower intake of potassium and vitamin C;<br>- Signif. lower intake of fruits rich in vitamin C and potassium
[A]de Franca, NAG, 2020 [21] Dietary intake, muscle strength, sedentary lifestyle	Brazil; Community dwelling; Health Survey of the City of São Paulo. (ISA-Capital 2015) (2015 ISA-Nutrition)	C-S Inclusion/ Exclusion criteria applied	T-score ≤-1 for hip and/or spine to include osteopenia and osteoporosis With DXA	ALM/BMI F <0.512 M <0.789 With DXA	FMI M>9 kg/m^2; F>13 kg/m^2 with DXA	Total, n=218; F, n=113 (52); M, n=105 (48); older adults, n=161 (74)	Mean 63; Range 59–69	Total, n=14 (6.4) F and M combined	DXA 24-h recall; Handgrip with Jamar® dynamometer; Gait speed usual pace, 4 m/min	Normal + 6 groups: osteopenia/ osteoporosis; sarcopenia; obesity; osteopenic sarcopenia; osteopenic obesity; sarcopenic obesity	- Signif. lower protein intake (g/kg/Wt) but not as % of energy;<br>-None of other nutrients were signif. different among groups;<br>- Signif. lower grip strength and more time spent sitting
[NoP]Cervo, MM, 2020 [25] Energy-adjusted Dietary inflammator	Australia: Population-based community dwelling; Southern	Prospective; with follow-up at 5 and 10 years;	Changes in T-score ≤-1 for hip and/or spine to include	Changes in ALM whole-body DXA; Hand grip strength;	Baseline BF%: F ~40 M ~28	Total at baseline, n=1098: F, n=562 (51);	Mean at baseline: 63; Range 51-79	Not reported; For every unit increase in E-DII	DXA, FFQ to calculate E-DII scores; Dynamometers for changes	With baseline values and changes at five and 10 years of follow-up	-Consumption of pro-inflammatory diet (higher E-DII scores), increased incidence of fractures over 10 years in M, but not in F,

y index (E-DII)	Tasmania, TASOAC 2002-2004	Inclusion/ Exclusion criteria applied	osteopenia & osteoporosis; With DXA	Knee extension; fall risks	With whole-body DXA BMI kg/m^2: F ~28 M ~ 27.7	M, n=536 (49); At 5 years, n=768; At 10 years, n=566		score, Incidence fracture increased 9% in M but decreased 12% in F	in grip strength and knee extension; PPA for changes in fall risk; Self - assessment questionnaires for fractures		despite being associated with reductions in lumbar spine and total hip BMD in both sexes; -E-DII scores signif. associated with higher fall risk scores and lower ALM in M but not in F.
Park S, 2018 [22] Dietary inflammatory index (DII); Higher scores denote higher proinflamma-tory diet	S. Korea, KNHANES, 2009-2011	C-S Retro-spective Inclusion/ Exclusion criteria applied	T-score ≤-1 for hip and/or spine to include osteopenia & osteoporosis; With DXA	ALM/weight <1SD of reference population; with DXA	BMI: kg/m2 based on Asian-Pacific guidelines overweight ≥23<25, obese ≥25	Total, n=1344 F only	Mean 62.3; OSO 64	Total, n=455 (31.8)	DXA, 24-h recall, DII score	Normal, osteosarco-penia, osteopenic obesity, sarcopenic obesity	-DII scores signif. associated with higher risk for OSO; -Groups with osteosarcopenia, osteopenic obesity, sarcopenic obesity had signif. lower intake of vitamins C and E compared to the normal group
Kim J, 2017 [15] Diet Quality-Index-International (DQI-I); higher scores denote better food quality intake	S. Korea KNHANES 2008-2010	C-S Retro-spective Inclusion/ Exclusion criteria applied	T-score ≤-1 (for Asian reference population) With DXA	ALM/Wt <1SD of Korean reference population (20-39 y) With DXA	BF% ≥40 of body fat by gender With DXA	Total, n=6129; F, n=3550; M, n=2579	F 61.9; M 60.8; OSO F 64.3; OSO M 64.2	F 25%; M 13.5%	DXA, 24-h recall	Healthy Korean adults aged 20–39 years	-In F: Higher scores on the DQI-I associated with better body composition phenotypes; -Signif. less intake of fish, mushrooms, milk, energy, protein -Tendency to less intake of meat, eggs; -In M: DQI-I scores were not associated with body composition abnormalities

[1]OSA/OSO terms are used interchangeably and reflect those used in each article. [2]Prevalence includes both pre- (with osteopenia and/or presarcopenia) and full OSA/OSO; [3]OSA/OSO participants were compared to those with one or more body composition impairments (e.g., osteopenia/osteoporosis, osteosarcopenia, sarcopenia, sarcopenic obesity, obesity/adiposity alone), or normal. [NoP]Studies Not reporting/calculating the Prevalence of OSA/OSO, but still analyzing three body composition compartments from which OSA/OSO prevalence and/or its relation to exposure variables could be derived. *Studies with the same population, design, and intervention, but different independent/exposure variables. [A]Studies reporting association with physical Activity or sedentary lifestyle, in addition to nutrition. Abbreviations: COVID: coronavirus disease; C-S: cross-sectional; T-score for bone mineral density (BMD g/cm2); BIA-ACC: Bioelectrical Impedance Analysis with BioTekna®; S-score for muscle; BF: Body Fat; NHR: Nursing home residents; F: females; M: males; DXA: Dual Energy Absorptiometry; BMD: Bone Mineral Density; BP: Blood Pressure; TC: Total Cholesterol; ApoA1: Apolipoprotein A1; ApoB: apolipoprotein B; LDL-C: Low Density Lipoprotein Cholesterol; HDL-C: High Density Lipoprotein Cholesterol; KNHANES: Korea National Health and Nutrition Examination

Survey; SMI: Skeletal Muscle Index (kg/height2); ALM: Appendicular Lean Mass; BMI: Body Mass Index; FMI: Fat Mass Index (body fat/height2); TASOAC: Tasmanian Older Adult Cohort Study; E-DII score: Dietary Inflammatory Index; FFQ: Food Frequency Questionnaire; PPA: Physical Profile Assessment; DQI-I: Quality Index-International.

Table S2. Characteristics and results of studies with serum nutritional biomarkers as independent variables related to osteosarcopenic adiposity/obesity (OSA/OSO)[1]

Reference, Studied topic	Country Setting	Study Design	Diagnostic criteria & Instruments			Sample size n (%)	Age (years)	OSA/OSO Prevalence[2] (%)	Assessment Tools	Compared to[3]	Outcomes in OSA/OSO group (or others if indicated)
			Bone	Lean/Muscle	Adipose						
Chung, S-J, 2022 [27] Serum ferritin; Subjects stratified by serum ferritin tertiles	S. Korea, Medical health screening and check-up	C-S Two-center; Inclusion/ Exclusion criteria applied	T-score ≤-1 for hip and/or spine to include osteopenia & osteoporosis; With DXA	SMI <1SD of reference population; With BIA	BF%: F ≥35; M ≥25 With DXA	Total, n=25,546; F, n=16,912; M, n=8634	Mean, 58.7; F, 58.3; M, 59.6; F-OSO 66.3; M-OSO 67.7	Total, 7.9%; F, 6.4%; M, 9.4%	DXA; InBody-720; Cobas 8000 (for ferritin), Roche Diagnostics	Normal; combinations: osteoporosis, and/or sarcopenia and/or obesity	-Higher serum ferritin signif. associated with combined adverse body composition in F, but not in M; -F in the highest ferritin tertiles had the highest OSO prevalence
[NoP]Ma, Y, 2020 [28] 25(OHD); Subjects stratified by 25(OH)D tertiles	China Nine province s,(comm unities)	C-S Inclusion/ Exclusion criteria applied	T-score ≤-1 for hip and/or spine to include osteopenia & osteoporosis With DXA	ALM; <1SD than mean; F 13.9 kg M 20.2 kg With DXA	BF%: F 36 M 27.5 With whole-body DXA	Total, n=4506; F, n=2905 (64.5); M, n=1601 (33.5)	Mean: 68.1; F, 67.6 M, 68.6	Not reported	DXA; Liquid chromatogra phy–tandem mass spectrometry (for 25(OH)D)	Osteopenic obesity, Sarcopenic obesity, Obesity-only	-25(OHD) deficiency associated with greater likelihood of OSO; -Independent negative dose-response associations of 25(OHD) with OSO and other impaired body composition components
[A]Kim, YM, 2019 [29] Serum 25(OH)D	S. Korea KNHAN ES V, 2008-2011	Retro-spective; Inclusion/ Exclusion criteria applied	T-score ≤-1 for hip and/or spine to include osteopenia & osteoporosis With DXA	ALM/Weig ht <1SD of reference population With DXA	BF%: F ≥35; M ≥ 25	Total, n=3267; F, n=2187; M, n=1080	Mean 64.2; F 63.8; M 64.6; F-OSO 66.3; M-OSO 67.7	Total 36.1%; F, 40.1%; M, 28.1%	DXA; Radioimmun o assay (DiaSorin) with 1470 Wizard γ-counter	Osteopenic obesity, Sarcopenic obesity, Obesity-only	-Both F-OSO and M-OSO had signifi. lower serum 25(OH)D (<20 ng/mL); -Both F and M engaged in the lowest physical activity; -F-OSO had the highest prevalence of hypertension, diabetes and metabolic syndrome
Kim, J, 2017 [30] Serum 25(OHD)	S. Korea KNHAN ES IV, 2008-2010	C-S Retro-spective; Inclusion/ Exclusion criteria applied	T-score ≤-1 (for Asian reference population) With DXA	ALM <1SD of ref. population With DXA	BF% ≥ 40 of body fat by gender With DXA	Total, n=5908; F, n=3423; M, n=2485	Mean 61.2; F 61.7; M 60.7; F-OSO 64.2; M-OSO 63.9	Total, 19.3%; F, 25%; M, 13.5%	DXA; DiaSorin (for 25(OH)D); 24-h recall	Osteopenic obesity, Sarcopenic obesity, Obesity -only	-Signif. higher prevalence of 25(OH)D (<20 ng/mL) in both F and M; -Higher 25(OH)D in mid- and later life signif. associated with reduced odds of adverse body composition, leading to OSA (stronger in M)

[1] OSA/OSO terms are used interchangeably and reflect those used in each article. [2]Prevalence includes both pre- (with osteopenia and/or presarcopenia) and full OSA/OSO; [3]OSA/OSO participants compared to those with one or more body composition impairments (e.g., osteopenia/osteoporosis, osteosarcopenia, sarcopenia, sarcopenic obesity, obesity/adiposity alone), or normal. [NoP]Studies not reporting/calculating the prevalence of OSA/OSO, but still analyzing three body composition compartments from which

OSA/OSO prevalence and/or its relation to exposure variables could be derived. [A]Studies reporting association with physical activity or sedentary lifestyle, in addition to nutrition. C-S: cross-sectional; T-score for bone mineral density (BMD, g/cm^2); DXA: Dual Energy Absorptiometry; SMI: Skeletal Muscle Index ($kg/height^2$); BIA-ACC: Bioelectrical Impedance Analysis with BioTekna®; BF: Body Fat; F: females; M: males; 25(OHD): 25-hydroxyvitamin D; ALM: Appendicular Lean Mass; KNHANES: Korea National Health and Nutrition Examination Survey.

Table S3. Characteristics and results of studies with physical activity as independent variables related to osteosarcopenic adiposity/obesity (OSA/OSO)[1]

Reference, Studied topic	Country, Setting	Study Design	Diagnostic criteria & Instruments			Sample size (n), Intervention	Age (years)	Prevalence[2] n (%)	Assessment Tools	Compared to[3]	Outcomes in OSA/OSO group (or others if indicated)
			Bone	Lean/Muscle	Adipose						
Lee, Y-H, 2021 [31] Progressive resistance training (peRET) effects on functional performance and body composition	Taiwan, Community dwelling women	Inclusion/ Exclusion Criteria applied; 12-week intervention with 2 randomized groups; Blinded randomization into groups	T-score ≤-1 for spine to include osteopenia & osteoporosis; With DXA	SMI <5.67 kg/m^2; AND grip strength <20 kg; OR gait speed <0.8 m/s	BF%: ≥35	Total, n=27; peRET, n=15; 40 min, three times/w; OR Control, n=12; No dropouts; >85% exercise compliance; Follow-up at 6 months	Mean 70.9; No diff. among groups	All participants, as per inclusion criteria	DXA; BIA Dynamometer, Thera-Band®	Baseline values; Control group of OSO women (attended group lectures with educational material)	-Signif. increase in BMD and T-score for spine -Signif. improvement in Functional Forward Reach; Timed up-and-go test; Timed chair-rise test; Gait speed; -No change in BF%, and some lean tissue parameters; -No sustainable benefits after 6 months follow-up
Shen, LI, 2020 [36] Aerobic exercise and resistance training combined effects on body composition	China, Community dwelling, women and men	Inclus/Exclusion Criteria applied; 12-week intervention with 2 randomized groups; No mention on assessor blinding	T-score ≤-1 to include osteopenia & osteoporosis; With DXA	SMI F ≤5.4 kg/m^2; M ≤7.0 kg/m^2	BF%: F ≥35; M ≥25	Total, n=30; Exercise, n=15; 45-60 min/day, 3 times/weak; OR Control, n=15;	>60 No diff. between groups	All participants, as per inclusion criteria	DXA; BIA Dynamometer, Elastic band	Control group of OSO women and men	-Signif. increase in BMD and decrease in BF%; - No change in SMI
[NoP]Cunha, PM, 2018 [32] Resistance training volume (1 & 3 sets) effects on bone, muscle and body fat	Brazil, Community dwelling, women	Inclusion/ Exclusion Criteria applied; 12-week intervention with 3 randomized groups;	No specific identification for bone, muscle and body fat status. Composite OSO Z-score derived from average of the muscular strength, SMM, % body fat, and BMD components was calculated by formula: (muscular strength Z-score)+(SMM Z-			Total, n=62; Intervention groups: 1-set training (n=21, for 15 min); OR	Mean 67.4; No diff. among groups	Not reported	DXA; Repetition Maximum (RM) by chest press, knee extension, preacher curl exercise	Baseline values; Also, 1 set vs. 3 sets of training; Control group	-Signif, increase in total strength; SMM; -Signif improvement in OSO Composite Z-score from baseline to-post test -Signif, decrease in body fat;

		Blinded randomization into groups	score)+(−1xbody fat Z-score)+ (BMD Z-score)/4			3-sets (n=20, for 50 min) 3-times/w; OR Control (n=21); ≥85% exercise compliance					-No change in BMD -Dose response to higher activity (3 sets induced higher improvement than 1 set); -Both sets induced higher improvement compared to control
Banitalebi, E,* 2020 [35] Elastic band resistance training effects on body composition, functionality, various OSO biomarkers	Iran, Community dwelling women	Inclusion/ Exclusion Criteria applied; 12-week intervention with 2 randomized groups; Concealed randomization (based on age and OSO composite Z-scores) into groups; Blood samples blinded for analysis	T-score ≤-1 for hip and/or spine to include osteopenia & osteoporosis; With DXA	10 m walk test ≤ 1 (m/s); SMI ≤ 28% OR ≤ 7.76 kg/m^2	BF%: ≥32 BMI: >30 kg/m^2	Total, n=63; Progressive Elastic Band resistance training up to 60 min. (3 times/week), n=32; OR Control, n=31; Intention to treat analysis; 85% exercise compliance; 19% & 29% dropout in exp. and control groups, respectively; 25% participants reported side effects in first 3 sessions	Range 60-80; Mean 64.1 No difference between groups	All participants, as per inclusion criteria	DXA; Dynamometer Thera-Band® ELISA for blood tests	Control group of OSO women	-Signif. increase in handgrip strength, timed chair-rise test, muscle quality; -Signif. increase in estradiol and decrease in leptin; -Slight improvement in OSO composite Z-score; -No difference in BMD; BF; SMI; gait speed and timed-up-and-go test
Banitalebi, E,* 2021 [33] Elastic band resistance training effects on OSO markers, serum microRNAs											-Slight but insignificant improvement in OSO serum and other markers; -Serum microRNAs (miR-133 & miR-206) changes correlated with changes in FRAX scores, serum 25(OH)D and alkaline phosphatase
Banitalebi, E,* 2023 [34] Elastic band resistance training effects on cardiometabolic risk factors											-Signif. decrease in ipid-accumulation product; Triglyceride-glucose-BMI index; Visceral adiposity index; Atherogenic index of plasma; Framingham risk score;

		-NO change in Triglycerides; Triglyceride-glucose index; triglyceride-glucose-waist circumference index; C-reactive protein; Metabolic syndrome severity score
Hashemi, A,* 2020 [37] Elastic band resistance training effects on vascular aging, serum microRNA-146	Total, n=48; Training, n=26 OR Control, n=22; Intention to treat analysis; 85% exercise compliance	-Signif. decrease in serum miR-146; total cholesterol, LDL<br>-Signif. increse in HDL;<br>-NO difference in body weight, BMI, BMD, C-reactive protein
Kazemipour, N* 2022 [38] Elastic band resistance training effects on IGF-1 and FGF-2		-Signif. increase in IGF-1 and FGF-2<br>NOT significant: Relationship of IGF-1 and FGF-2 with BMD<br>-NO change in BMD

[1] OSA/OSO terms are used interchangeably and reflect those used in each article. [2]Prevalence includes both pre- (with osteopenia and/or presarcopenia) and full OSA/OSO; [3]OSA/OSO participants compared to those with one or more body composition impairments (e.g., osteopenia/osteoporosis, osteosarcopenia, sarcopenia, sarcopenic obesity, obesity/adiposity alone), or normal. [NoP]Studies not reporting/calculating the prevalence of OSA/OSO, but still analyzing three body composition compartments from which OSA/OSO prevalence and/or its relation to exposure variables could be derived. *Studies with the same population, design, and intervention, but different independent/exposure variables. T-score for bone mineral density (BMD g/cm^2); DXA: Dual Energy Absorptiometry; SMI: Skeletal Muscle Index (kg/height^2); BF: Body Fat; BIA: Bioelectrical Impedance Analysis; F: Female; M: Male; SMM: Skeletal Muscle Mass; BMI: Body Mass Index; ELISA: Enzyme-Linked Immunosorbent Assay; FRAX- score: Fracture Risk Assessment; 25(OH)D: 25 hydroxyvitamin D (calcitonin); IGF-1= insulin growth factor; FGF-2- fibroblast growth factor; LDL: Low Density Lipoproteins; HDL: High Density Lipoproteins.
